# A putative bifunctional CPD/ (6-4) photolyase from the cyanobacteria *Synechococcus* sp. PCC 7335 is encoded by a UV-B inducible operon: New insights into the evolution of photolyases

**DOI:** 10.3389/fmicb.2022.981788

**Published:** 2022-10-28

**Authors:** María Belén Fernández, Lucas Latorre, Natalia Correa-Aragunde, Raúl Cassia

**Affiliations:** Instituto de Investigaciones Biológicas, Facultad de Ciencias Exactas y Naturales, Universidad Nacional de Mar del Plata, Consejo Nacional de Investigaciones Científicas y Técnicas, Mar del Plata, Argentina

**Keywords:** cryptochrome/photolyase family, *Synechococcus* sp. PCC 7335, UV-B, photolyase operon, cyanobacteria, bifunctional CPD/(6-4) photolyase- like

## Abstract

Photosynthetic organisms are continuously exposed to solar ultraviolet radiation-B (UV-B) because of their autotrophic lifestyle. UV-B provokes DNA damage, such as cyclobutane pyrimidine dimers (CPD) or pyrimidine (6-4) pyrimidone photoproducts (6-4 PPs). The cryptochrome/photolyase family (CPF) comprises flavoproteins that can bind damaged or undamaged DNA. Photolyases (PHRs) are enzymes that repair either CPDs or 6-4 PPs. A natural bifunctional CPD/(6-4)- PHR (PhrSph98) was recently isolated from the UV-resistant bacteria *Sphingomonas* sp. UV9. In this work, phylogenetic studies of bifunctional CPD/(6-4)- photolyases and their evolutionary relationship with other CPF members were performed. Amino acids involved in electron transfer and binding to FAD cofactor and DNA lesions were conserved in proteins from proteobacteria, planctomycete, bacteroidete, acidobacteria and cyanobacteria clades. Genome analysis revealed that the cyanobacteria *Synechococcus* sp. PCC 7335 encodes a two-gene assembly operon coding for a *PHR* and a *bifunctional CPD/(6-4) PHR- like*. Operon structure was validated by RT-qPCR analysis and the polycistronic transcript accumulated after 15 min of UV-B irradiation. Conservation of structure and evolution is discussed. This study provides evidence for a UV-B inducible PHR operon that encodes a CPD/(6-4)- photolyase homolog with a putative bifunctional role in the repair of CPDs and 6-4 PPs damages in oxygenic photosynthetic organisms.

## Introduction

Ultraviolet- B (UV-B) is the solar electromagnetic radiation with wavelengths between 280–315 nm. Although most of this radiation is absorbed by the stratospheric ozone layer it affects all living organisms. Around 0.3% of sunlight energy at sea level corresponds to UV-B and it can damage aquatic organisms triggering a decrease of ecosystem productivity (Häder et al., 2011; Banaś et al., 2020). UV-B is perceived by the UV-B response locus 8 (UVR8) photoreceptor present in photosynthetic organisms ranging from green algae to higher plants (Fernández et al., 2016). Although no defined photoreception systems have been described for other photosynthetic microorganisms (red and brown algae and cyanobacteria), they have developed several mechanisms of protection against UV-B damage (Montgomery, 2007; Sinha and Häder, 2008; Rastogi et al., 2014).

Cyanobacteria are an ancient group of Gram-negative bacteria, and the first prokaryotes that perform oxygenic photosynthesis. Cyanobacteria are ubiquitous and occupy diverse ecological niches, adapting to various extreme environments, such as high or low temperatures, highly acidic or basic pH, high salt concentrations, desiccation and UV-B (Wada et al., 2013). As photosynthetic organisms, they depend on solar energy and have to cope with harmful UV-B. Solar UV-B affects the DNA and protein structures, photosynthesis, ribulose 1,5-bisphosphate carboxylase/oxygenase (RuBisCO) activity, N_2_ fixation, cellular morphology, growth, survival, pigmentation and buoyancy (Tyagi et al., 1992; Rastogi et al., 2014; Kumar et al., 2020; Vega et al., 2020). Thus, cyanobacteria developed several UV protection mechanisms that include UV absorbing and screening compounds such as scytonemin and mycosporine-like amino acids (MAAs), antioxidant protection, apoptosis, migration, mat formation and DNA reparation to recover UV induced DNA damages (Sinha and Häder, 2008; Moon et al., 2012; Rastogi et al., 2014).

Solar UV radiation can induce two types of pyrimidine dimers in the double helix DNA, being the predominant cyclobutane pyrimidine dimers (75%; CPDs) and to a lesser extent pyrimidine (6-4) pyrimidone photoproducts (6-4 PPs) (Banaś et al., 2020; Vechtomova et al., 2020). Photoreactivation is a blue/ UV-A light- dependent mechanism used to specifically repair CPD or 6-4 PPs damages by photolyases (PHRs) (Pathak et al., 2019; Soumila Mondal et al., 2022). These enzymes are a class of flavoproteins found in all organisms excluding placental mammals, who lost all genes encoding functional photolyases in the course of evolution (Banaś et al., 2020; Xu et al., 2021). During repair, flavin adenine dinucleotide (FAD) cofactor is fully reduced *via* Trp or Tyr surface transfer of electrons in a process named photoreduction (Graf et al., 2015). Then, a rapid electron transfer from the excited fully reduced FAD chromophore to the DNA lesion triggers both kinds of repair. The 6-4 PPs repair also requires proton transfer which is a limiting step resulting in much lower reaction efficiency (Banaś et al., 2020). A bifunctional photolyase called as PhrSph98 was recently characterized in the Antarctic bacterium *Sphingomonas* sp. UV9. This enzyme is able to repair both types of damage since it displays a larger catalytic pocket compared to CPDs or 6-4 PPs repairing enzymes (Marizcurrena et al., 2020).

The cryptochrome/photolyase family (CPF) comprises proteins that have conserved the FAD binding site and switch between basal and excited states. CPF proteins bind either damaged or undamaged DNA and are classified in two groups according to their function: (1) CPD and 6-4 PPs photolyases, which repair CPD or 6-4 PPs damages, respectively, and (2) Cryptochromes (CRY) that regulate growth and development in plants and the circadian clock both in plants and animals (Mei and Dvornyk, 2015; Vechtomova et al., 2020).

CPD PHRs are classified, based on sequence similarity in (i) class 0, which repair CPD damages in single-stranded DNA (ssDNA PHR, previously classified as CRY with Cry-DASH designation), (ii) class I, present mostly in unicellular organisms; (iii) class II, in unicellular and multicellular organisms; and (iv) class III found only in some eubacteria (Ozturk, 2017; Zhang et al., 2017). Recently, an exhaustive phylogenetic analysis identifies a new class of CPD repair enzymes, called short photolyase-like (SPL), because they lack the N-terminal α/β domain of normal photolyases. They are similar to class I/III CPD PHRs and authors speculated that this class constitutes the real ancestor of the CPF (Xu et al., 2021). CPD PHRs III may be considered as an intermediate form between CPD PHR I and plant CRYs, as well as a protein that has retained the traits of their common ancestor (Vechtomova et al., 2020). The iron–sulfur cluster containing bacterial cryptochromes and 6-4 PPs repair photolyases (FeS –BCPs) are newly characterized CPF members containing the amino acids necessary to bind cofactors, and four conserved cysteine residues for the coordination of an iron -sulfur cluster, an ancient feature (Scheerer et al., 2015).

Some CPFs have dual roles, like ssDNA PHR proteins. They are restricted to UV lesions and repair CPD damages in single-stranded DNA, having also blue light photoreceptor activity. These proteins may represent the link between photolyases and cryptochromes (Kiontke et al., 2020). It has been proposed that the bifunctional CPD/(6-4)- PHR from *Sphingomonas* sp. UV9, PhrSph98, is a sister group of CPD class II PHRs and that may represent a missing link in the transition from 6-4 PP to CPD PHRs (Marizcurrena et al., 2020).

It is widely accepted that photolyases are ancient DNA repair enzymes, which have evolved far before the oxygen accumulation and the ozone layer establishment in the atmosphere (Vechtomova et al., 2020). However, the evolutionary scenario was not fully elucidated yet. Prokaryotic 6-4 photolyases were suggested as the first common ancestor of photolyases. However, as CPDs are the major UV-induced DNA damage it is not convincing that the 6-4 photo repair occurred earlier than the CPD one during evolution (Xu et al., 2021). Exhaustive phylogenetic trees do not include the recently described bifunctional CPD/(6-4)- photolyase PhrSph98, and its occurrence among other species was not explored. Thus, the aim of this work was (1) to analyze the distribution of CPD/(6-4) photolyase- like proteins and its evolutionary relationship with other CPF family members; and (2) characterize the operon structure that encodes a *bifunctional CPD/(6-4) PHR- like* gene from the cyanobacteria *Synechococcus* sp. PCC 7335. We show that PhrSph98 homologs are present both in heterotrophic and phototrophic bacteria and that cyanobacteria *Synechococcus* sp. PCC 7335 encodes this homolog, in a two-gene assembly operon, which is induced by UV-B light. Evolutionary and structural aspects of this operon are discussed.

## Materials and methods

### Defined criteria for the homology search

A protein–protein BLAST (BlastP) analysis was performed against the non- redundant protein sequence (nr) database using as template the amino acid sequence of PhrSph98 from *Sphingomonas* sp. UV9 (accession number: NCBI ANW48627), and restricting the maximum target sequences to 250 to increase taxon- specific occurrence. All the retrieved sequences match the criteria of E- values lower than 0.001 (being lower than 1×10^−63^) and percentage identity higher than 30% according to Pearson (2013). Subsequently, partial sequences and those sharing >85% of identity (redundant proteins) were identified and discarded using CD-HIT software (Huang et al., 2010).^1^

### Phylogenetic analysis and protein conserved domain search

Multiple sequence alignment and curation was performed using MAFFT^2^ and BMGE 1.12_1 (Criscuolo and Gribaldo, 2010)^3^ software, respectively, using default settings. Subsequently, the phylogenetic tree was inferred using a total length of the alignment of 259 aminoacids with PhyML 3.0^4^ using the automatic model selection AIC, which defined LG5 + G + I + F as the best model of evolution, and 1,000 bootstrap. The tree was visualized using iTOL (Letunic and Bork, 2021).^5^

A sequence alignment logo for the proteins described as homologs of PhrSph98 was created using the online available WebLogo tool^6^ (Crooks et al., 2004).

Conserved domains were identified using the sequences from each target protein employing the NCBI conserved domain database (CDD)-NIH^7^ (Lu et al., 2020).

### Operon prediction and sequence analysis

Operon prediction was performed using the webservers Operon mapper^8^ (Taboada et al., 2018) and SoftBerry^9^ restricting the search to bacterial genomes.

Operon promoter and potential transcription factors binding sequences were predicted using BPROM software from SoftBerry platform.^10^ Transcriptional terminator sequences were predicted using iTerm-PseKNC/predictor^11^ (Feng et al., 2019).

### Structure prediction of putative bifunctional CPD/(6-4)- PHR from *Synechococcus* sp. PCC 7335

The linear amino acid sequence of PhrSph98 was used as template in BlastP against the nr protein database restricting the search to *Synechococcus* sp. PCC 7335. Tertiary structure of PhrSph98 and *Synechococcus* sp. PCC 7335 homolog were predicted using Modeller in the HHPred server^12^ (Söding et al., 2005). The structure of class II CPD-photolyase from *Methanosarcina mazei* (PDB ID: 2XRY) was the most similar to both queries, and thus selected as template. The 3D model structures were validated using ProSA web software (Wiederstein and Sippl, 2007). Visualization, structure overlaps and distance calculations were done using the Molecular Graphics System PyMOL.

### Bacterial strain and culture method

The cyanobacterial strain *Synechococcus* sp. PCC 7335 used in this study was acquired from the Pasteur Culture Collection of Cyanobacteria.^13^ *Synechococcus* sp. PCC 7335 was originally isolated from a snail shell in an intertidal zone near Puerto Peñasco, Mexico (Rippka et al., 1979). PHR genes were identified in the genome of *Synechococcus* sp. PCC 7335 (NCBI accession number NZ_DS989904.1). Cultures were grown in Erlenmeyer flasks in volumes of 150 ml containing ASNIII marine medium at 25°C and photon flux density of 5 μmol m^−2^ s^−1^ under a photoperiod of 12 h light (4,500 K LED tubes): 12 h dark. The optical density at 750 nm (OD750 nm) was measured with GeneQuant 1,300 spectrophotometer to monitor cell growth.

### UV-B treatment

*Synechococcus* sp. PCC 7335 cells were grown in at least three replicate cultures to an OD750 nm ~0.2–0.4. Aliquots of cells (~35 ml) were collected from each replicate culture, placed in Petri dishes and exposed to 3.34 μmol m^−2^ s^−1^ of UV-B supplemented with ~5 μmol m^−2^ s^−1^ of PAR for 15 and 30 min in a controlled environment chamber. The UV-B irradiance intensities were chosen according to He et al. (2021). Control samples were covered with a polycarbonate filter of 1 mm to screen UV-B. UV-B was provided by narrowband UV-B Philips TL 100 W/01 lamps. The spectral irradiance was determined with an UV- B photo-radiometer (Delta ohm HD2102.1).

After treatment, cells were immediately pelleted by centrifugation at 11,000× *g* and 4°C and stored at −80°C until being processed for total RNA extraction.

### RNA extraction and RT-qPCR analysis

RNA from *Synechococcus* sp. PCC 7335 was extracted with the RNeasy mini Kit (Qiagen) following the manufacturer’s protocol. The concentration and purity of the isolated RNA were measured with a UV spectrophotometer (NanoDrop™ One, Thermo Scientific). Total RNA was used for cDNA synthesis in a reaction containing 3 μM random primer, 10 mM dNTP, 0.1 M DTT, and 200 U of M-MLV reverse transcriptase (Invitrogen).

RT-qPCR was performed at a 10 μl total reaction volume including 1 μl of a dilution of cDNA template, 250 nM of forward and reverse primers, and 5 μl of 2× Power SYBR Green PCR Mix (Applied Biosystem). Amplified signals were monitored continuously with a Step One Plus Real-Time Thermal Cycler (Applied Biosystem). Thermocycling was performed using the following conditions: 10 min of denaturation and enzyme activation at 95°C, followed by 40 cycles at 95°C for 15 s and 60°C for 1 min. Melting curve analysis was performed from 60 to 95°C at 0.3°C increments to verify primer specificity. Negative controls (no template and minus RT transcriptase) were included in every PCR run to verify no genomic DNA contamination. A default threshold of 1 was used. The size of qPCR products for each primer pair was verified on 2% (w/v) agarose gel electrophoresis using 1× TAE buffer. Ladder 100 pb was used as molecular marker (PB-L productos Bio- lógicos). Real- time data was analyzed using the StepOne™ Software v2.3 Tool (Applied Biosystem). The primers used are listed in Supplementary Table S1. RNase P RNA (*RNPB*) and Phosphoenolpyruvate carboxylase (*PPC*) were used as reference genes for gene expression normalization. Results were expressed as 2^(−∆Ct)^ being ∆Ct the ratio between the target gene and the geometric mean of *PPC* and *RNPB* (Livak and Schmittgen, 2001).

## Results

### BLASTp analysis and amino acid conservation

**Figure 1:**
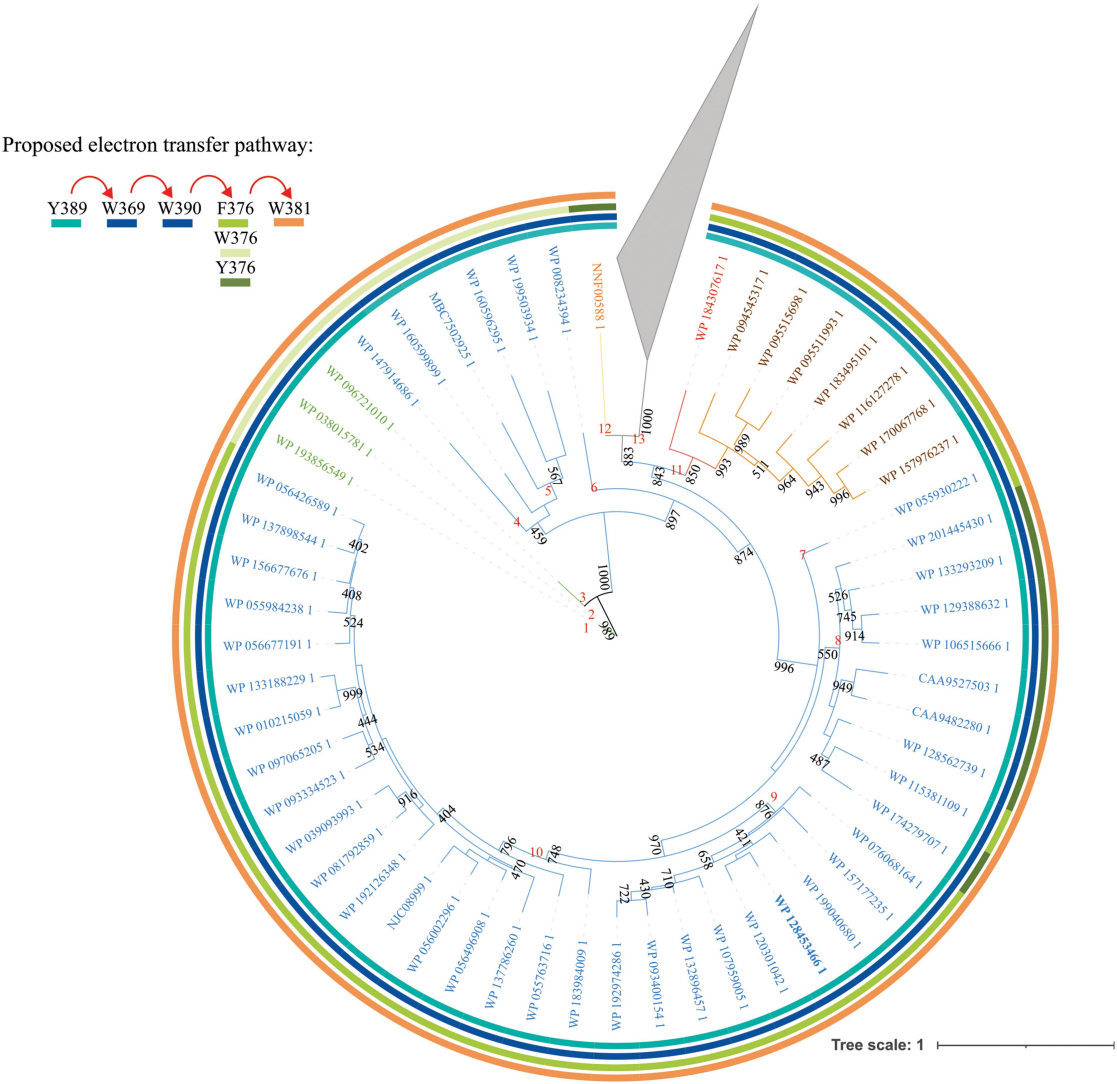
Unrooted maximum- likelihood tree of bifunctional CPD/(6-4) PHR- like proteins. Protein sequences with similarity to PhrSph98 from *Sphingomonas* sp. UV9 were retrieved using BLASTp and the non-redundant database. Representative sequences were used for further analysis. Evolutionary history was inferred using the maximum- likelihood method based on LG + G+ I+ F model using PHYML 3.0 (Guindon et al., 2010). The phylogenetic tree is presented as unrooted. Nonparametric bootstrapping (1,000 replicates) was used to assess tree branching support and is shown adjacent to each internal node. Those lower than 400 are not shown. Rings of different colors indicate the amino acids described in PhrSph98 to be potentially involved in electron transfer pathway: Y389- W369- W390- W376- W381. Red arrows indicate the electron flux. Text in color indicates clade of the predicted homologs of PhrSph98: green, cyanobacteria; blue, proteobacteria; red, planctomycete; brown, bacteroidete; orange, acidobacteria. The accession number of each sequence is shown. Proteins without homology in amino acids involved in PhrSph98 activity are collapsed (gray triangle). Numbers in red indicate different nodes. Full phylogenetic tree is shown in Supplementary Figure S1.

In order to analyze phylogenetic positioning of the CPD/(6-4)- PHR from *Sphingomonas* sp. UV9 (PhrSph98) we performed a BLASTp search of the NCBI non-redundant protein database. Results retrieved 250 sequences (Supplementary Table S2) with similarities ranging from 90 to 31% and E-values from 0 to 1.10^−63^ matching the criteria of homology reported previously (Pearson, 2013). MSA and phylogenetic analysis showed that the closest homologs to PhrSph98 were grouped in a clade (numbered as 7, 8, 9 and 10, Figure 1) containing proteins with E-values of 0 and percentages of identity ranging from 61.32 to 82.04 (Supplementary Table S3). These proteins correspond to Gram- negative bacteria, including *Sphingomonas* as the most abundant (31/37), and others like *Belnapia* (1/37), *Dankookia* (1/37), *Sphingosinicella* (1/37), *Methylobacterium* (1/37), *Polymorphobacter* (1/37) and *Croceibacterium* (1/37) (Supplementary Table S3). According to the domain profile search using the NCBI conserved domain database (CDD)- NIH, the majority of these proteins contain the PHR2 domain, except for two proteins from node 9 that have a PHRB domain (WP_157177235.1 from *Sphingomonas prati* and WP_093400154.1 from *Sphingomonas jinjuensis*, Supplementary Figure S1).

#### Amino acids involved in electron transfer

**Figure 2:**
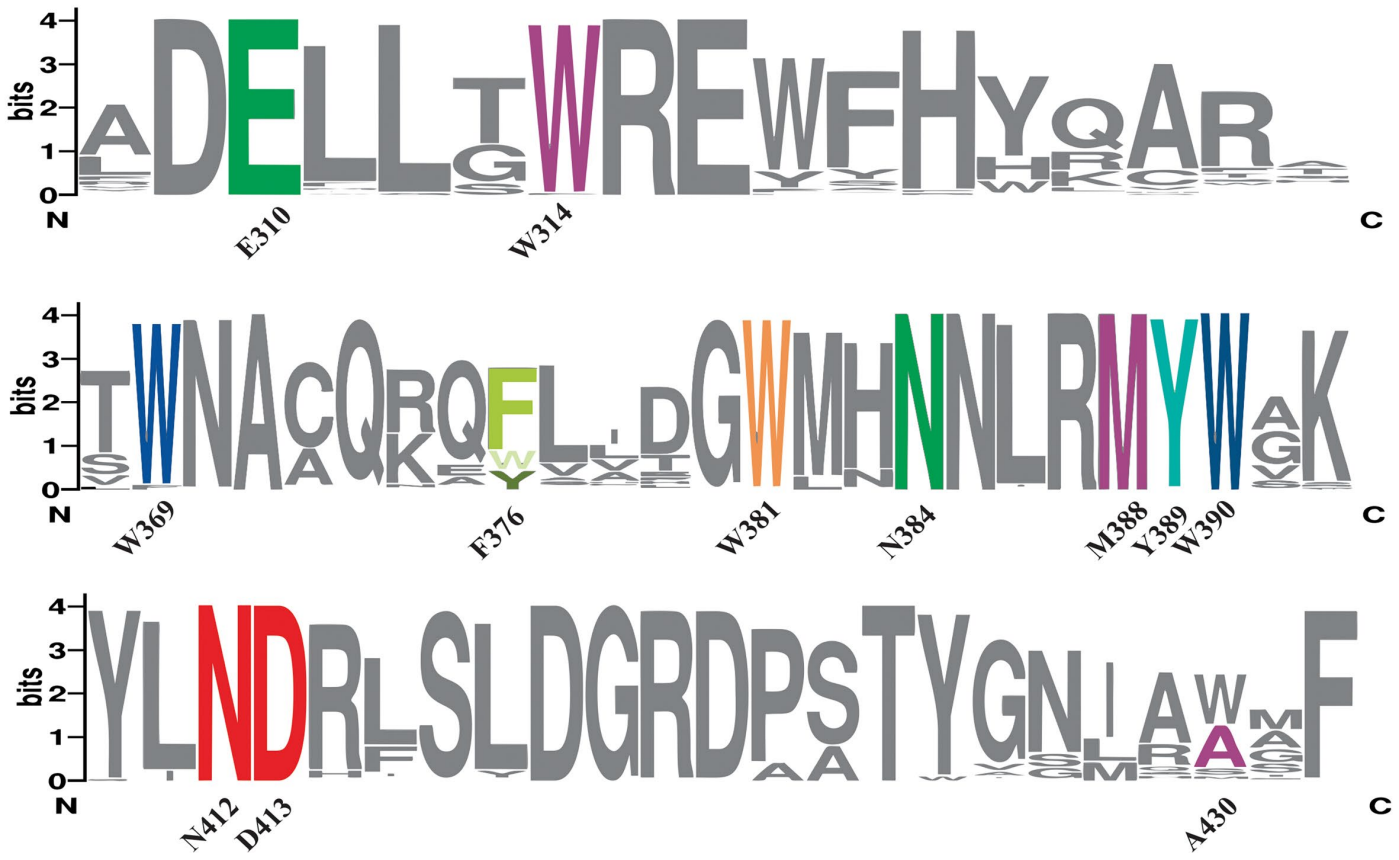
Sequence conservation of predicted bifunctional CPD/(6-4) PHRs. MSA from predicted bifunctional photolyases was used to create a sequence alignment Logo showing the conservation of amino acids involved in electron transfer (Y389-W369-W390-F376-W381, in the same colors as phylogeny rings from Figure 1), FAD binding (N412, D413 in red), lesion binding (W314, A430, M388 in purple) and lesion stabilization (E310, N384 in green). The height of each letter is proportional to the frequency of the corresponding amino acid and the overall height of each stack for a position is proportional to the sequence conservation, measured in bits. N at the left and C at the right indicate amino- and carboxyl- terminal regions. Numbers indicate amino acid position in PhrSph98.

*In silico* proposed 3D model from PhrSph98 suggests that electron transfer to FAD involves Y389-W369-W390-F376-W381-FAD pathway (Marizcurrena et al., 2020). We analyzed the conservation of amino acids involved in electron transfer using a MSA. Figures 1 and 2 show that W369 and 390 were conserved in the 149 sequences analyzed. Y389 is conserved from nodes 1 to 12, being changed for a Thr or Leu (which could not participate in the electron transfer) in the species from node 13 (Figure 1; Supplementary Figure S1). F376 was conserved in nodes 7, 9, 10 and 11 and in WP_115381109 from *Sphingomonas* sp. strain FARSPH in node 8 (Figure 1). This amino acid was replaced for a Trp in nodes 1 to 6, and for a Tyr in nodes 8 and 12 (Figure 1). In the other predicted proteins, there was a change to non- aromatic amino acids in this position (Supplementary Figure S1). Finally, W381 was conserved from nodes 1 to 12 (Figure 1). Thus, aromatic amino acids important for electron transfer and DNA repair are conserved in species from nodes 1 to 12, suggesting that they constitute PhrSph98 homologs.

#### FAD binding domain

In class II CPD PHRs, N403 is highly conserved and involved in FAD cofactor binding (Kiontke et al., 2011). This residue from *M. mazei* class II photolyase (template for PhrSph98 tertiary structure modeling) corresponds to N412 in PhrSph98 (Marizcurrena et al., 2020). MSA analysis showed that this residue was conserved in all the sequences predicted as homologous by phylogeny (Figure 2). A charge compensation during FADH- photoreduction is performed by H- bonding of N403 and the surface exposed D404 in *M. mazei* CPD PHR (Kiontke et al., 2011). This residue (D413 in PhrSph98) was also conserved in PhrSph98 homologs (Figure 2) indicating that FAD binding is favored in these enzymes.

#### Amino acids involved in DNA lesion binding

CPD and 6-4 PPs DNA lesions are predicted to be positioned in the binding pocket of PhrSph98 through interaction with the hydrophobic residues W314, A430 and M388 (Marizcurrena et al., 2020). W314 was conserved among all the proteins identified as PhrSph98 homologs (Figure 2). In nodes 1 to 8 and 11 to 13 the position of A430 was replaced for a Trp, as occurs in *M. mazei* CPD photolyase II (W421) (except for WP_095511993.1, WP_095515698.1 from node 11 and TWT53083.1 from node 13). In nodes 9 and 10, A430 was conserved except for WP_157177235.1, WP_107959005.1, WP_133188229.1, WP_010215059.1, WP_056426589.1. M388 was conserved among all the protein sequences analyzed, except for protein TWT53083.1 (Figure 2).

#### Amino acids involved in lesion stabilization

Amino acids E310 and N384 from PhrSph98 are hypothesized to be involved in the stabilization of (i) the CPD radical after electron transfer (by the transfer of a proton from a neutral Glu at the bottom of the active site) and (ii) the anionic thymine radical after bond breakage (through hydrogen bond formation with the N3 amide and C4 carbonyl from CPD lesion) respectively (Marizcurrena et al., 2020). Both amino acids were conserved in the sequences predicted to have bifunctional CPD/(6-4)- activities (Figure 2).

Considering these results, over a total of 149 sequences we propose that 55 are true homologs of PhrSph98 (proteins from nodes 1 to 12, Figure 1), as they conserve all the important amino acids described to be involved in electron transfer, cofactor and DNA lesion binding and stabilization (Figure 2). A widespread distribution of these enzymes was observed among bacteria, including different genera from proteobacteria (43/55), planctomycete (1/55), bacteroidetes (7/55), acidobacteria (1/55) and cyanobacteria (3/55) (Figure 1) being the latter the only oxygenic photosynthetic organisms encoding this enzyme.

### Phylogeny of CPF proteins

**Figure 3:**
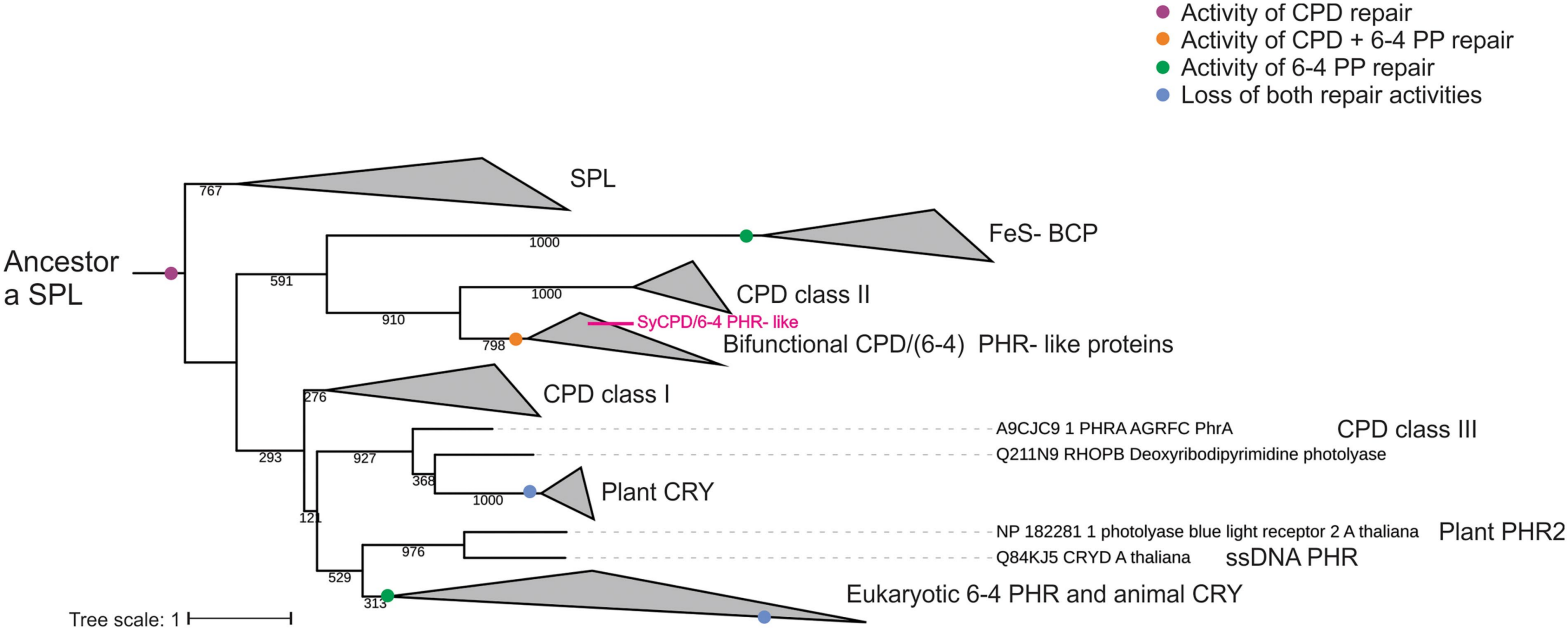
Phylogenetic distribution of the cryptochrome/photolyase family including bifunctional CPD/(6-4) PHR- like proteins. Phylogenetic tree with a CPD photolyase with an iron–sulfur cluster (SPL) as the first common ancestor. Proposed major evolutionary events regarding DNA damage repair activity are indicated by colored dots. Maximum-likelihood probabilities of 1,000 replicates are adjacent to each internal node. Branches corresponding to proteins from the same clade were collapsed. Complete phylogeny is shown in Supplementary Figure S4.

The phylogenetic tree obtained from the CPF proteins was unrooted, with an apparent root on the branch near the group of FeS-BCPs. We manually placed the branches from FeS-BCPs, SPL and bifunctional photolyases as roots in independent trees (Supplementary Figure S3). It was found that the simplest tree which has minimum changes across evolution correspond to SPL as root. The maximum likelihood tree reconstruction features 10 main clades. It comprises SPL, CPD PHRs class I, II and III, plant CRY, plant PHR2, ssDNA PHRs, animal CRY and eukaryotic (6-4) photolyases, FeS-BCPs and bifunctional CPD/(6-4) PHR- like proteins (Figure 3, see Supplementary Figure S4 for full phylogenetic tree).

All bifunctional CPD/(6-4) PHR- like proteins constituted one monophyletic group, which was located as a sister group of class II CPD PHRs. This clade was located as a sister group of FeS-BCPs enzymes and all together as sister group of all the rest of the members from the CPF (Figure 3). According to tree inference, FeS-BCPs and eukaryotic 6-4 photolyases lost the CPD activity and gained 6-4 PP photorepair activity through two independent events, whereas bifunctional CPD/(6-4) PHR -like proteins gained 6-4 PP activity but retained CPD repair activity (Figure 3). Results suggest that bifunctional CPD/(6-4) PHR- like proteins are related to class II CPD PHRs, both sharing a common ancestor with FeS-BCPs, in a clade that includes only prokaryotic organisms.

### Sequence homology of PhrSph98 and cyanobacteria proteins: Structure modeling of bifunctional CPD/(6-4) photolyase- like protein from the cyanobacteria *Synechococcus* sp. PCC 7335

**Figure 4:**
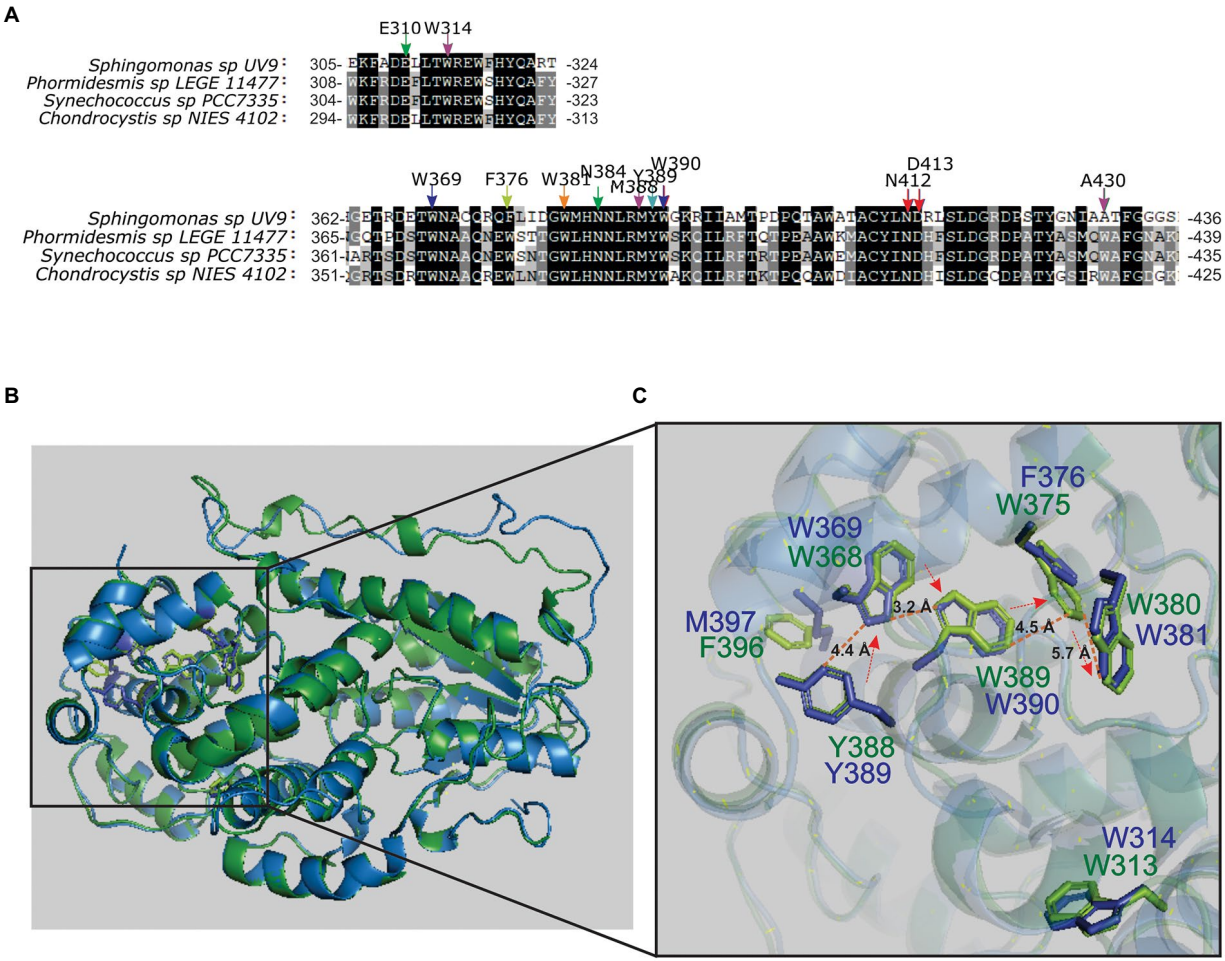
Multiple sequence alignment and molecular modeling in cyanobacteria. **(A)** MSA from PhrSph98 protein and cyanobacteria homologs was performed using MAFFT software. Arrows indicate conserved amino acids involved in electron transfer pathway (in the same colors as phylogeny rings from Figure 1), FAD cofactor binding (red), DNA lesion binding (purple) and lesion stabilization (green). Conserved residues common to all sequences are shadowed in black and less identity is shown in gray scale. Full MSA is presented in Supplementary Figure S2. **(B)** Superposition of predicted crystal tertiary structures from PhrSph98 (blue) and *Synechococcus* sp. PCC 7335 bifunctional CPD/(6-4) photolyase- like protein (green). **(C)** Amplification of catalytic domain. Amino acids described that may be involved in electron pathway in PhrSph98 are shown in blue Y389-W369-W390-F376-W381. Homologs from *Synechococcus* sp. PCC 7335 bifunctional CPD/(6-4) PHR- like protein are shown in green. The arrows indicate the electron flux direction. Dash lines indicate distances in amstrongs (Å) among atoms. **(B)** and **(C)** were generated by using PyMOL.

Phylogenetic analysis revealed that canonical CPD/(6-4) PHR- like proteins are also present in a few strains of oxygenic photosynthetic organisms. Blast analysis using PhrSph98 as query toward the *Viridiplantae* group gave no results, indicating that the only oxygenic photosynthetic organisms encoding a bifunctional photolyase were the cyanobacteria *Phormidesmis* sp. *LEGE 1147*, *Chondrocystis* sp. NIES-4102 and *Synechococcus* sp. PCC 7335 (Figure 1; Supplementary Figure S1). Amino acids involved in DNA repair, lesion and cofactor binding were conserved among PhrSph98 and the cyanobacterial homologs (Figure 4A).

*Synechococcus* sp. PCC 7335 has several adaptations to different ambient conditions as chromatic acclimation (CA), far- red light photoacclimation (FARLIP) and the presence of a non- canonical nitric oxide synthase enzyme (SyNOS) (Ho et al., 2017; Correa-Aragunde et al., 2018; Herrera-Salgado et al., 2018). In this work, we were particularly interested in the strategies of *Synechococcus* sp. PCC 7335 to cope with UV-B radiation. To analyze that, tertiary structure of PhrSph98 homolog from *Synechococcus* sp. PCC 7335 (herein named as SyCPD/(6-4) PHR- like protein) was predicted using as template a class II CPD-photolyase from the archaea *M. mazei* (PDB ID: 2XRY) using HHPred online available server. Results obtained show that PhrSph98 and SyCPD/(6-4) PHR- like protein (identity of 40.71%) adopted similar tertiary structure (Figure 4B). Molecular modeling indicate that the positioning of the amino acids Y389-W369-W390-F376-W381 were conserved in SyCPD/(6-4) PHR- like protein (Figure 4B) suggesting it may be a true bifunctional CPD/(6-4)- PHR.

### The *bifunctional CPD/(6-4) PHR- like* gene in *Synechococcus* sp. PCC 7335 is part of an UV-inducible operon

Genomic context analysis revealed that *SyCPD/(6-4) PHR- like* gene constitutes a two gene operon assembly with another PHR (herein refer as PHRs operon). This operon is only conserved in *Phormidesmis* sp. LEGE 11477 (accession JADEXO010000055.1, 81.68% of identity and 95% of query coverage) not in *Chondrocystis* sp. NIES-4102. This study provides evidence of a bifunctional CPD/(6-4) PHR- like protein, as well as a putative PHRs operon in an oxygenic photosynthetic organism.

**Figure 5:**
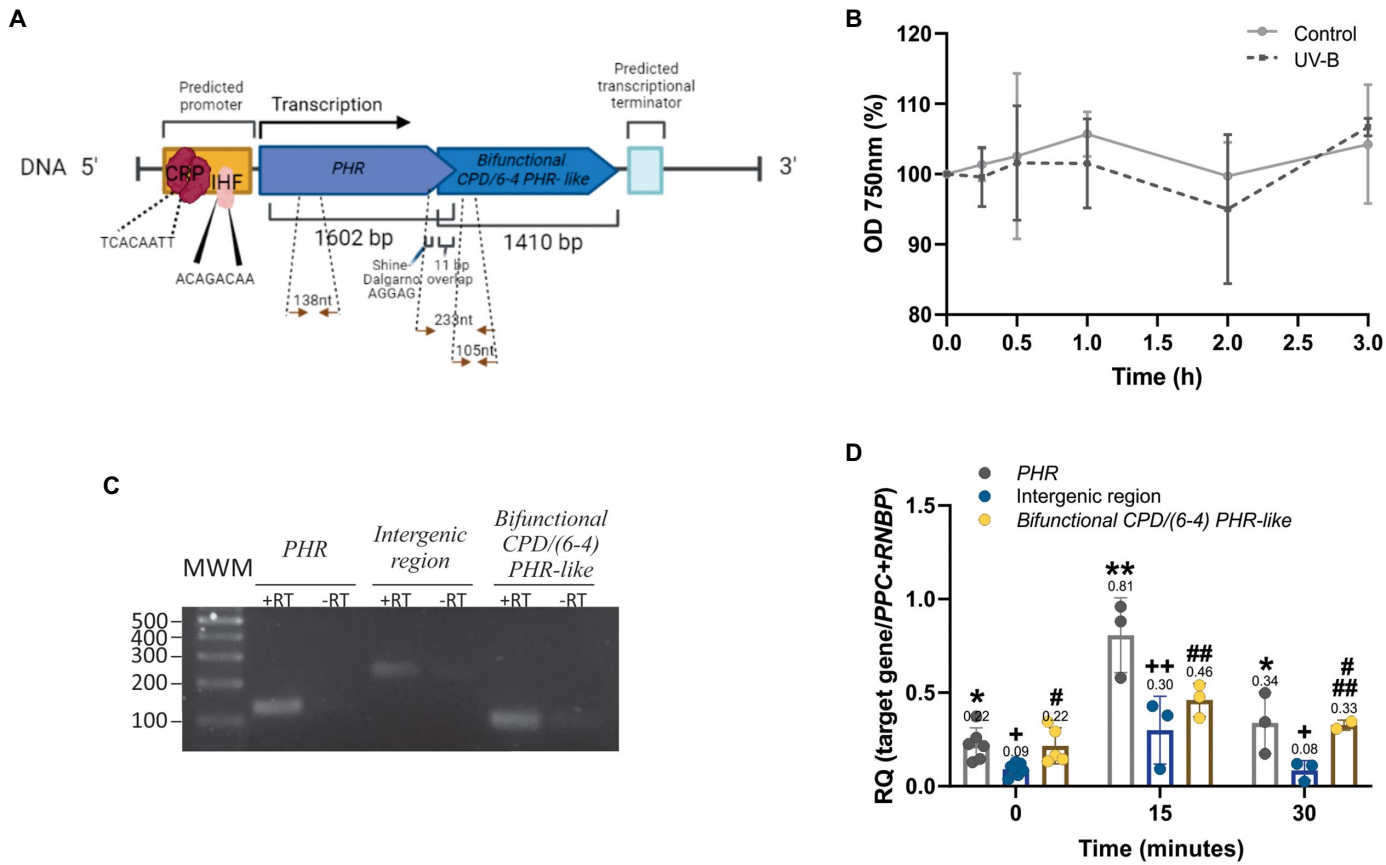
Structure and expression of the PHRs operon from *Synechococcus* sp. PCC 7335. **(A)** Gene organization scheme of the PHRs operon. Arrows indicate the position of the primers used for RT- qPCR. Nucleotide sequence from the PHRs operon is shown in Supplementary Figure S5. **(B)** Growth curves of *Synechococcus* sp. PCC 7335 strain under control or UV-B treatment recorded as optical density (OD) at 750 nm during 3 h. **(C)** Amplicons from *PHR*, *bifunctional CPD/(6-4) PHR- like* and the intergenic region (+RT) were loaded on agarose 2% (w/v) and subjected to electrophoresis. Negative controls without reverse transcriptase (−RT) were included in the assay. MWM: molecular weight markers. **(D)** PHRs expression under UV-B treatment. The transcript level of *PHR* (grey), *SyCPD/(6-4) PHR- like* (yellow) and the intergenic region (blue) of control and UV-B exposed cultures were evaluated by RT-qPCR. Results were expressed as 2^-(∆Ct)^ using as normalizer the geometric mean of *PPC* and *RNBP* genes. Normalization of gene expression to individual reference genes is shown in Supplementary Figure S6. The One- way ANOVA test was used to determine differences for PHR (*), the intergenic region (+) and *bifunctional* CPD/(6-4) PHR- like (#) transcript levels at different time treatments (*p* < 0.05). Equal symbols represent no statistical differences.

PHRs operon is encoded in the DNA negative strand (coding sequence from position 38,587 to 35,587). Two putative −10 (TGCTATACA) and −35 (TTGAAG) boxes were found in the promoter region using the bacterial promoter prediction software BPROM. The analysis of potential transcription factor binding sites revealed the sequences TCACAATT from cyclic AMP receptor protein (CRP) and ACAGACAA from integration host factor (IHF) (Figure 5A). The operon consists of two unidirectional structural genes: an upstream deoxyribodipyrimidine photo-lyase (PHR, WP_038015784.1) and a downstream hypothetical protein (SyCPD/(6-4) PHR- like, homolog to PhrSph98, WP_038015781.1) with different translation frames (Figure 5A). Both ORFs overlap in 11 nt within the sequence **ATG**TCAGT**TGA** where TGA is the stop for the upstream gene, and ATG is the start of the second (Figure 5A). We also found a Shine- Dalgarno (SD) intragenic sequence (AGGAG) preceding in seven nucleotides the ATG from the downstream gene. This sequence is absent in the leading gene (Figure 5A). Details of PHRs operon nucleotide sequence are shown in Supplementary Figure S5.

Photolyases transcript accumulation is induced upon UV-B exposition in several organisms, protecting them from cell death (An et al., 2021; Fernández et al., 2021, 2022; He et al., 2021). First, we analyzed the effect of UV-B irradiation (1.6 W m^−2^) on *Synechococcus* sp. PCC 7335 cell culture measuring the OD at 750 nm. As shown in Figure 5B, UV-B does not affect *Synechococcus* sp. PCC 7335 optical density at 750 nm up to 3 h- treatment.

To investigate whether the two PHRs ORFs form a polycistronic mRNA, the intergenic region between *PHR* and *SyCPD/(6-4) PHR- like* was amplified by qPCR from non- irradiated culture cDNA. Figure 5C shows the amplification of the expected DNA fragment (233 nt), confirming the expression of both PHRs as a polycistronic mRNA. The expression of *PHR*, *SyCPD/(6-4) PHR- like* and the intergenic region were also evaluated during UV-B treatment. Results show that *PHR*, *bifunctional CPD/(6-4) PHR- like* and the intergenic region expression increased 3.5, 2 and 3- fold, respectively, after 15 min of UV-B exposition (Figure 5D). PHR transcript was statistically significantly higher compared to bifunctional photolyase. After 30 min of UV-B exposure, abundance of all amplicons decreased to control levels (Figure 5D).

## Discussion

### Bifunctional CPD/(6-4) PHR- like proteins domain structure is conserved among heterotrophic bacteria and cyanobacteria

Photolyases repair the UV-induced DNA damages of CPDs and 6-4 PPs using blue/ UV-A light. The FAD catalytic cofactor, conserved in the whole protein superfamily of photolyase/cryptochromes, adopts a unique folded configuration at the active site and plays a critical role in DNA repair. FADH- functions as the active state to allow efficient electron injection into DNA damage in a process that involves intramolecular electron transfer (Zhang et al., 2017). Based on the *in silico* modeling of PhrSph98 from *Sphingomonas* sp. UV9, Marizcurrena et al. (2020) suggested that electron transfer to FAD involves Y389-W369-W390-F376-W381 amino acids pathway. In class II CPD photolyases, an Asn residue (N403) is highly conserved and involved in FAD cofactor binding. Kiontke et al. (2011) showed that the class II CPD photolyase from *M. mazei* owing N403A and N403L mutations has impaired FAD binding. In contrast, the N403D mutant has FAD incorporation of at least 70% (Kiontke et al., 2011). Also, in class II CPD PHRs, a charge compensation during FADH- photoreduction is performed by H- bonding of the side chain from N403 with D404, whereas in class I this role is taken up by a Glu residue (Kiontke et al., 2011). We showed that all these residues are conserved in PhrSph98 homologs, indicating that bifunctional CPD/(6-4) PHR- like proteins are present in heterotrophic bacteria like proteobacteria, planctomycete, bacteroidete, acidobacteria and in oxygenic photosynthetic bacteria. Conservation of class II CPD PHRs residues suggests that these enzymes belong to this class, which was also supported by phylogenetic analysis, positioning bifunctional photolyases as a sister group of CPD class II photolyases.

Additionally, tertiary structure model comparison between PhrSph98 and SyCPD/(6-4) PHR- like protein shows that the structural arrangement of residues involved in electron transfer to FAD, FAD binding, and DNA damages binding are conserved. The tertiary structure conservation of PhrSph98 hallmarks, allows us to suggest that SyCPD/(6-4) PHR- like is a true homolog of this enzyme.

### Bifunctional CPD/(6-4) PHR- like proteins clade is a sister group of class II CPD PHRs

Several ancestors for the CPF family have been proposed (Mei and Dvornyk, 2015; Miles et al., 2020; Vechtomova et al., 2020). After analyzing different phylogenetic tree topologies, we found that positioning of SPL proteins as root retrieves the tree with lesser changes during evolution. Accordingly, Xu et al. (2021) suggested that the first common ancestor of the CPF might be a SPL protein, with CPD photolyase activity and a FeS cluster, characteristic of ancient proteins. The phylogenetic tree shows that bifunctional CPD/(6-4) PHR- like proteins are a sister group of CPD class II photolyases and both constitute a clade with FeS-BCP proteins, represented only by prokaryotic organisms. 6-4 PP photorepair appear independently in FeS-BCP and bifunctional proteins, probably by mutational events, and conservation of CPD activity is only retained in bifunctional enzymes. A 6-4 PP repair enzyme can be converted into a CPD repair one with only three mutations. However, eleven mutations are needed for the vice versa conversion (Yamada et al., 2016). This asymmetric functional conversion was suggested as a more complex repair mechanism for 6-4 PPs repair (Yamada et al., 2016). Although it seems that conversion of CPD to 6-4 PP activity is more difficult in terms of the number of mutations, the phylogenetic tree supports the origin of the CPF with a CPD repair family.

### Characterization of *Synechococcus* sp. PCC 7335 photolyase operon

An operon is a cluster of neighboring genes that are transcribed together and therefore encodes several proteins. Its evolution is hypothesized to be adaptive and toward coordinated optimization of functions (Memon et al., 2013). Results presented here indicate that only the cyanobacteria *Synechococcus* sp. PCC 7335 and *Phormidesmis* sp. LEGE 11477 encode a PHRs operon. It has recently been proposed a new classification of the genus *Synechococcus*, based on habitat distribution patterns (seawater, freshwater, brackish and thermal environments) that reflect the ecological and evolutionary relationships of its members. According to Salazar et al. (2020), *Synechococcus* sp. PCC 7335 genome did not cluster with any other *Synechococcus* genome, and matched with the Phormidesmiales order, being named *Phormidesmis mexicanus* PCC 7335. This new genus assignment is supported by the conservation of PHRs operon both in *Phormidesmis* sp. LEGE 11477 and *Synechococcus* sp. PCC 7335. Thus, the *Phormidesmis* genus is apparently the unique containing a PHRs operon including a bifunctional CPD/(6-4) PHR- like protein. Currently, there is a low number of publicly available cyanobacteria genomic sequences (0.6% compared to the total number of genomes available for bacteria and archaea; Alvarenga et al., 2017). Future genome sequencing of this phylum will allow us to determine whether this operon occurs in other cyanobacteria.

Operon evolution analysis in cyanobacteria shows that genes in highly and moderately conserved operons code for key cellular processes, such as photosynthesis. Conversely, genes in poorly conserved operons may code for functions possibly linked to niche adaptation. Also, newly acquired operons are greater in number, smaller in size, with wider intergenic spacing, and weakly coregulated compared to ancient operons. However, a sub-clade comprising the genera *Synechocystis*, *Microcystis*, *Cyanothece,* and *Synechococcus* sp. PCC 7002 forms an exceptional case with small intergenic spaces (Memon et al., 2013). *Synechococcus* sp. PCC 7335 PHRs operon matched the criteria of poorly conserved and no intergenic space, indicating that may be a newly formed operon with an adaptative role to protect this organism from high environmental UV-B levels. Here, we observed that UV-B induces the expression of the PHRs operon. This arrangement guarantees that the production of the proteins related to DNA repair under UV-B induced damage is simultaneously switched on and off and provides the ability to fast acclimate to new growth conditions. Moreover, there is a potential binding site of the transcription factor CRP upstream of the PHRs operon promoter −35 and −10 boxes. Class I CRP-dependent promoters have this sequence upstream of the promoter boxes. This transcriptional activation involves the interaction between CRP and the carboxy-terminal domain of the RNA-polymerase (RNA-P) α-subunit, facilitating the binding of RNA-P to the promoter (Soberón-Chávez et al., 2017). Additionally, a *CRP* knockout strain of *Deinococcus radiodurans* is sensitive to UV radiation, suggesting an important role of CRP in UV protection (Yang et al., 2016). Also, in *D. radiodurans*, a CRP homolog regulates the expression of different DNA repair proteins as RecN (in response to double-stranded DNA breaks), PprA (RecA-independent, DNA repair-related protein) and UvsE (UV damage endonuclease that is involved in nucleotide excision repair). Based on this, CRP transcription factor is an interesting candidate to be explored for PHRs operon UV-B inducible expression regulation.

Until now, only two reports describe photolyases encoded in operons. Osburne et al. (2010) characterized an operon of two DNA repair genes (nudix hydrolase- photolyase operon) in the UV hyper-resistant strain marine cyanobacterium *Prochlorococcus* MED4 which has constitutively upregulated expression but was induced by UV-B in the WT *Prochlorococcus* strain. Also, Sancar et al. (1984), reported an operon of a photolyase with a protein of unknown function in *E. coli*. To our knowledge, our study constitutes the first report that describes a UV-B inducible operon encoding two photolyases, being one of them with a putative bifunctional role in the repair of CPD and 6-4 PP damages. We found that upon 15 min of UV-B exposition, PHR transcript accumulates faster than bifunctional CPD/(6-4) PHR- like. The increase in transcripts level during UV-B irradiation, suggest a role in DNA repairing. The distinct expression of both intra-operonic genes constitutes a deviation from the generally expected co-expression behavior. A possible explanation may be the occurrence of translational coupling where the juxtaposition of the translational stop codon of an upstream gene with the translational start codon of the downstream one reduces the expression levels of the latter and affects mRNA stability (Dogra et al., 2015; Wright et al., 2021). In this sense, the upstream translating ribosome destabilizes RNA secondary structure which would prevent optimal expression of the downstream gene (Schümperli et al., 1982). We also found an intragenic SD sequence preceding the *bifunctional CPD/(6-4) PHR- like* gene, that may be involved in termination- reinitiation of translation. In overlapping genes, the start codon of the downstream gene is typically preceded by an SD motif in most archaea and bacteria (Huber et al., 2019). Cyanobacteria make ample use of the SD motif for reinitiation at overlapping gene pairs while less than 20% of SD sequences are found in the leading gene from operon (Huber et al., 2019). In agreement, no SD sequence was found in the leading gene of PHRs operon.

*Synechococcus* sp. PCC 7335 was isolated from a snail shell, from the intertidal zone in Puerto Peñasco, Mexico. As previously mentioned, they have several specific adaptations to prevailing light. For example, they contain chlorophyll *f* that absorbs far-red light and go under FaRLiP response and also perform complementary chromatic acclimation. They also contain an operon of PHRs that may contribute to UV-B tolerance, and one of these PHRs may encode for a bifunctional CPD/(6-4) PHR- like protein. Genome analysis of *Synechococcus* sp. PCC 7335 denotes the presence of two other CPF: a cryptochrome/photolyase family and a deoxyribodipyrimidine photo- lyase (WP_006457129.1, locus 27315-28829 and WP_006453873.1, locus 88140-86491 respectively). BlastP analysis of these two proteins shows similarity with cyanobacteria proteins. However, PHRs operon shares more similarities with proteobacteria photolyases. In this sense, it is possible that *Synechococcus* sp. PCC 7335 acquires the PHRs operon gene through horizontal gene transfer from proteobacteria. Whether these photolyases have redundant roles or contribute to classifying *Synechococcus* sp. PCC 7335 as an UV-B resistant bacteria needs to be explored. A metagenomic analysis combined with solar radiation measurements described a positive correlation between higher UV-B levels in the aquatic microbiome environments and the abundance of CPF genes (Alonso-Reyes et al., 2020). The finding of an operon of PHRs including a bifunctional CPD/(6-4)- photolyase- like protein may explain its ability to survive in an environment exposed to high doses of UV-B irradiation as are the intertidal zones (Hanelt et al., 1997). Additionally if bifunctional *CPD/(6-4) PHR- like* gene is under horizontal gene transfer is also an open question. Furthermore, it will be interesting to analyze whether the repair of two DNA damages by a single protein may confer on *Synechococcus* sp. PCC 7335 an advantage over expressing a CPD and 6-4 PP photolyases.

Exposure of humans to UV-B provokes DNA damage in keratinocytes that contributes to photoaging and implies a risk of progression to squamous cell carcinoma. Photolyases are not encoded by placental mammals. Photoprotection by the application of conventional sunscreens is not sufficient once DNA damage occurred. Diverse studies suggested that the addition of liposome-encapsulated CPD photolyases to sunscreens helps to diminish CPDs in human skin (Luze et al., 2020). Indeed, the biochemical characterization of cyanobacteria photolyases with potential bifunctional activity in the repair of both CPDs and 6-4 PPs may provide useful biotechnological information for the cosmetic and healthcare industries, as these organisms are emerging as sources of bioactive compounds.

## Data availability statement

The datasets presented in this study can be found in online repositories. The names of the repository/repositories and accession number(s) can be found in the article/Supplementary material.

## Author contributions

MBF conceived the original idea, carried out the phylogenies and experiments, and wrote the manuscript. LL collaborated in qPCR experiments. MBF, LL, NC-A, and RC contributed to the interpretation of the results. NC-A and RC contributed to the design of the experiments and critically revised the manuscript. RC supervised the project. All authors contributed to the article and approved the submitted version.

## Funding

This work was supported by the Agencia Nacional de Promoción Científica y Tecnológica (ANPCYT) grant number 2019-1577 to MBF, 2018-2524 to NC-A and 2019-3436 to RC, and Universidad Nacional de Mar del Plata grant number EXA 1030/21 to RC. MBF, NC-A, and RC are permanent researchers of CONICET Argentina. LL is PhD student fellow of CONICET.

## Conflict of interest

The authors declare that the research was conducted in the absence of any commercial or financial relationships that could be construed as a potential conflict of interest.

## Publisher’s note

All claims expressed in this article are solely those of the authors and do not necessarily represent those of their affiliated organizations, or those of the publisher, the editors and the reviewers. Any product that may be evaluated in this article, or claim that may be made by its manufacturer, is not guaranteed or endorsed by the publisher.

## Footnotes

^1^ http://weizhong-lab.ucsd.edu/cdhit-web-server/cgi-bin/index.cgi?cmd=cd-hit

^2^ https://mafft.cbrc.jp/alignment/server/

^3^ https://ngphylogeny.fr/tools/tool/273/form

^4^ http://www.atgc-montpellier.fr/phyml/

^5^ https://itol.embl.de/login.cgi

^6^ https://weblogo.berkeley.edu/logo.cgi

^7^ https://www.ncbi.nlm.nih.gov/cdd/

^8^ https://biocomputo.ibt.unam.mx/operon_mapper/

^9^ http://www.softberry.com/berry.phtml?topic=index&group=programs&subgroup=gfindb

^10^ http://www.softberry.com/

^11^ http://lin-group.cn/server/iTerm-PseKNC/pre.php

^12^ https://toolkit.tuebingen.mpg.de/tools/hhpred

^13^ http://www.pasteur.fr/
