# Supplementary material for: A putative bifunctional CPD/ (6-4) photolyase from the cyanobacteria Synechococcus sp. PCC 7335 is encoded by a UV-B inducible operon: New insights into the evolution of photolyases
Source: Front Microbiol. 2022 Oct 28;13:981788. doi: 10.3389/fmicb.2022.981788 (PMC9649867; doi:10.3389/fmicb.2022.981788)
Supplement: Supplementary file 1 [file Data_Sheet_1.docx]

Supplementary Material

A putative bifunctional CPD/ (6-4) photolyase from the cyanobacteria *Synechococcus* sp. PCC 7335 is encoded by a UV-B inducible operon: New insights into the evolution of photolyases

**Fernández María Belén*^1^, Latorre Lucas^1^, Correa-Aragunde Natalia^1^, Cassia Raúl^1^**

^1^Instituto de Investigaciones Biológicas- Facultad de Ciencias Exactas y Naturales, Universidad Nacional de Mar Del Plata- Consejo Nacional de Investigaciones Científicas y Técnicas, CC1245 7600, Mar Del Plata, Buenos Aires, Argentina.

*** Correspondence:**

Fernández María Belén

[mbfernan@mdp.edu.ar](mailto:mbfernan@mdp.edu.ar)

**Table S1. Sequences and molecular characteristics of the primers used in this study.**

| **Gene symbol** | **Accession No.** | **Official Full Name (MGI)** | **Primer Pair (5′–3′)** | **Amplicon length (bp)** | **Primer efficiency (%)** | **R^2^** | **Amplicon Tm (°C)** | **Reference** |
| --- | --- | --- | --- | --- | --- | --- | --- | --- |
| *PHR* | [WP_038015784.1](https://www.ncbi.nlm.nih.gov/protein/WP_038015784.1?report=genbank&log$=prottop&blast_rank=1&RID=0ABSNSPN013) | Deoxyribodipyrimidine photo-lyase | Fw GGCAGTATTTGTTCCTCAAGG | 138 | 90 | 0.99 | 78.22 | This paper |
|  |  |  | Rv GCTCGATATTGCCGTATGT |  |  |  |  |  |
| *Bifunctional CPD/(6-4)- PHR- like* | [WP_038015781.1](https://www.ncbi.nlm.nih.gov/protein/WP_038015781.1?report=genbank&log$=prottop&blast_rank=1&RID=0ABCNG18013) | Hypothetical protein | Fw CGGGTTCGCTCAAAGCAG | 105 | 89 | 0.99 | 77.52 | This paper |
|  |  |  | Rv GCTCGGTTGAGGATCACTA |  |  |  |  |  |
| Intergenic region | - | - | Fw AGGCTTCGGCTACTTCC | 233 | 88 | 0.99 | 79.72 | This paper |
|  |  |  | Rv GCTCGGTTGAGGATCACTA |  |  |  |  |  |
| *RNPB* | - | RNase P RNA | Fw CGGCTCAAAGCAAGGCTCAA | 123 | 93 | 0.99 | 79.45 | Correa- Aragunde et al., 2018 |
|  |  |  | Rv GATGCGATTACGGACGACTGC |  |  |  |  |  |
| *PPC* | WP_198011378.1 | Phosphoenolpyruvate carboxylase | Fw CACCCTGCCCGAATTATCGGTAC | 151 | 90 | 0.99 | 76.52 | This paper |
|  |  |  | Rv CCACGTAACGTCAGGAGTGACAG |  |  |  |  |  |


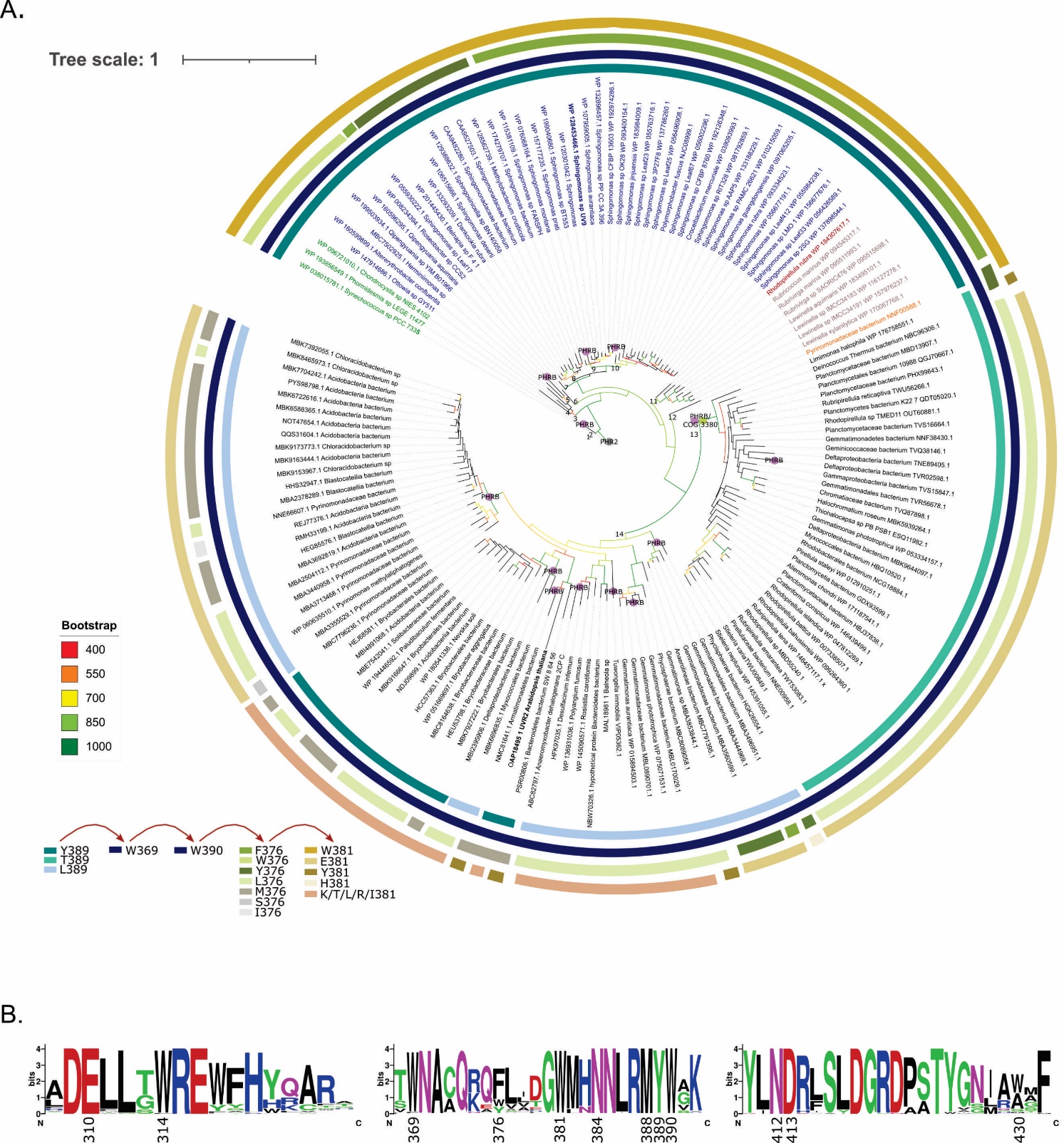


**Figure S1. Unrooted maximum- likelihood tree of bifunctional CPD/(6-4)- PHRs. A.** Protein sequences with similarity to PhrSph98 from *Sphingomonas* sp. UV9 were retrieved using BLASTp and the non-redundant database. CD-HIT software (Huang et al., 2010) was used to remove all sequences with identities >85% (redundant proteins). *Arabidopsis* UVR2 protein, is a class II CPD PHR that repairs CPD lesions upon UV-B irradiation and was included in the tree inference (Landry et al., 1997). The sequences were aligned with MAFFT (<http://mafft.cbrc.jp/alignment/server/>). Selection of phylogenetic informative regions from the multiple sequence alignment was performed using the BMGE 1.12_1 software (Criscuolo and Gribaldo, 2010). Maximum likelihood phylogenetic tree was calculated with LG +G+ I+ F model using PHYML 3.0 (Guindon et al. 2010) and is presented as unrooted. Nonparametric bootstrapping (1000 replicates) was used to assess tree branching support. The program iTol was used to display phylogenetic trees (<http://itol.embl.de/>) (Letunic and Bork, 2021). Rings of different colors indicate the amino acids described in PhrSph98 to be potentially involved in electron transfer pathway: Y389- W369- W390- W376- W381. Red arrows indicate the electron flux. Protein domains PHRB, PHR2 and COG3380 are shown in each node. Text in color indicate bacteria taxonomic classification of predicted homologs of PhrSph98: green, cyanobacteria; blue, proteobacteria; red, planctomycete; brown, bacteroidete; orange, acidobacteria. The accession number of each sequence is given next to the species name. Bootstrap values are shown in colors being red the lowest (400) and green the highest (1000). Bootstrap values lower than 400 are not shown.

**Table S3.** **List of closest homologs to PhrSph98.** Genus, accession numbers, E- values and percentage identity of the closest homologs to PhrSph98 grouped in clade containing branches numbered as 7, 8, 9 and 10 from phylogeny S1.

| Genus | Accession number | E- value | Identity (%) | Ratio |
| --- | --- | --- | --- | --- |
| *Sphingomonas* | WP_055930222.1  WP_106515666.1  CAA9482280.1  CAA9527503.1  WP_174279707.1  WP_115381109.1  WP_076068164.1  WP_157177235.1  WP_199040680.1  WP_120301042.1  WP_128453466.1  WP_107959005.1  WP_132896457.1  WP_192974286.1  WP_093400154.1  WP_183984009.1  WP_055763716.1  WP_137786260.1  WP_056496908.1  WP_056002296.1  WP_192126348.1  WP_039093993.1  WP_081792859.1  WP_133188229.1  WP_010215059.1  WP_097065205.1  WP_093334523.1  WP_056677191.1  WP_055984238.1  WP_156677676.1  WP_056426589.1 | 0  0  0  0  0  0  0  0  0  0  0  0  0  0  0  0  0  0  0  0  0  0  0  0  0  0  0  0  0  0  0 | 65.86  65.25  66.8  65.38  61.32  61.41  81.11  76.02  83.13  81.31  100  79.15  82.04  81.63  78.30  68.7  66.87  67.30  69.55  69.69  67.7  66.3  67.35  67.08  68.62  66.8  67.15  69.59  67.08  68.15  66.87 | 31/37 |
| *Belnapia* | WP_201445430.1 | 0 | 66.60 | 1/37 |
| *Dankookia* | WP_133293209.1 | 0 | 66.67 | 1/37 |
| *Sphingosinicella* | WP_129388632.1 | 0 | 66.67 | 1/37 |
| *Methylobacterium* | WP_128562739.1 | 0 | 64.84 | 1/37 |
| *Polymorphobacter* | NJC08999.1 | 0 | 71.13 | 1/37 |
| *Croceibacterium* | WP_039093993 | 0 | 66.3 | 1/37 |


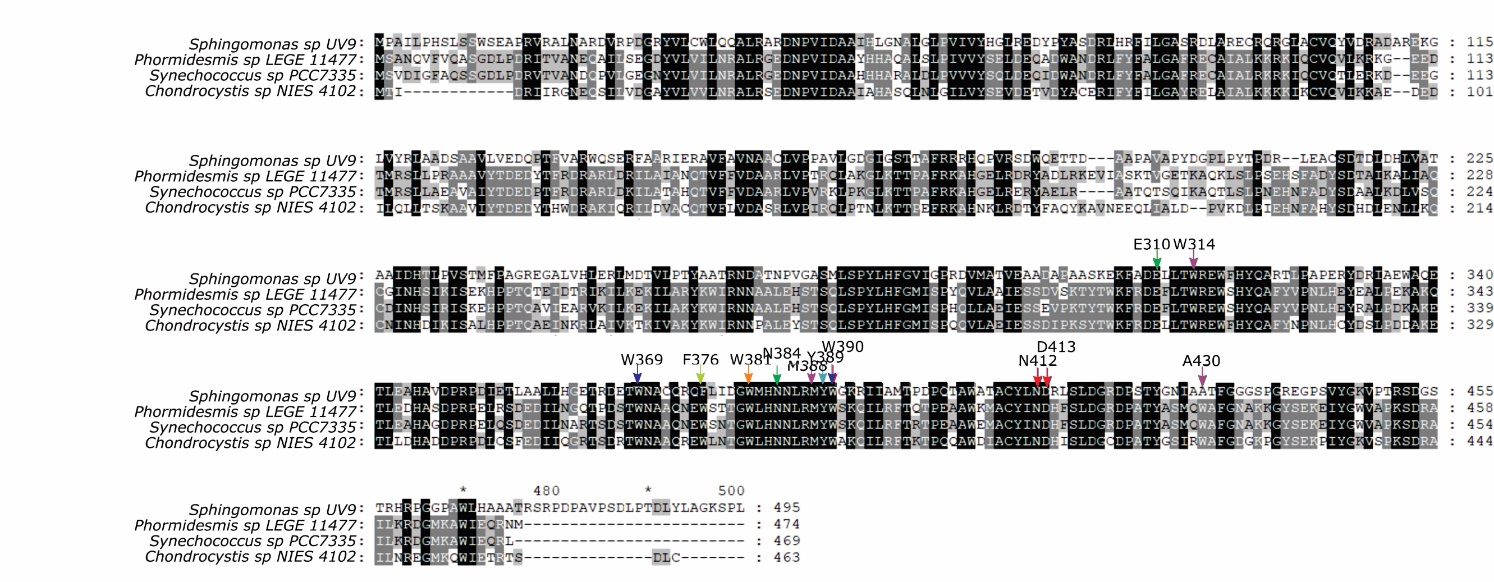


**Figure S2. MSA from *Sphingomonas* SV9 CPD/6-4 bifunctional photolyase homologs in cyanobacteria.** MSA was performed using MAFFT software. Arrows indicate conserved amino acids involved in electron transfer pathway (in the same colors as phylogeny rings from Figure S1), FAD cofactor binding (red), DNA lesion binding (purple) and lesion stabilization (green). Conserved residues common to all sequences are shadowed in black and less identity is shown in gray scale.

**
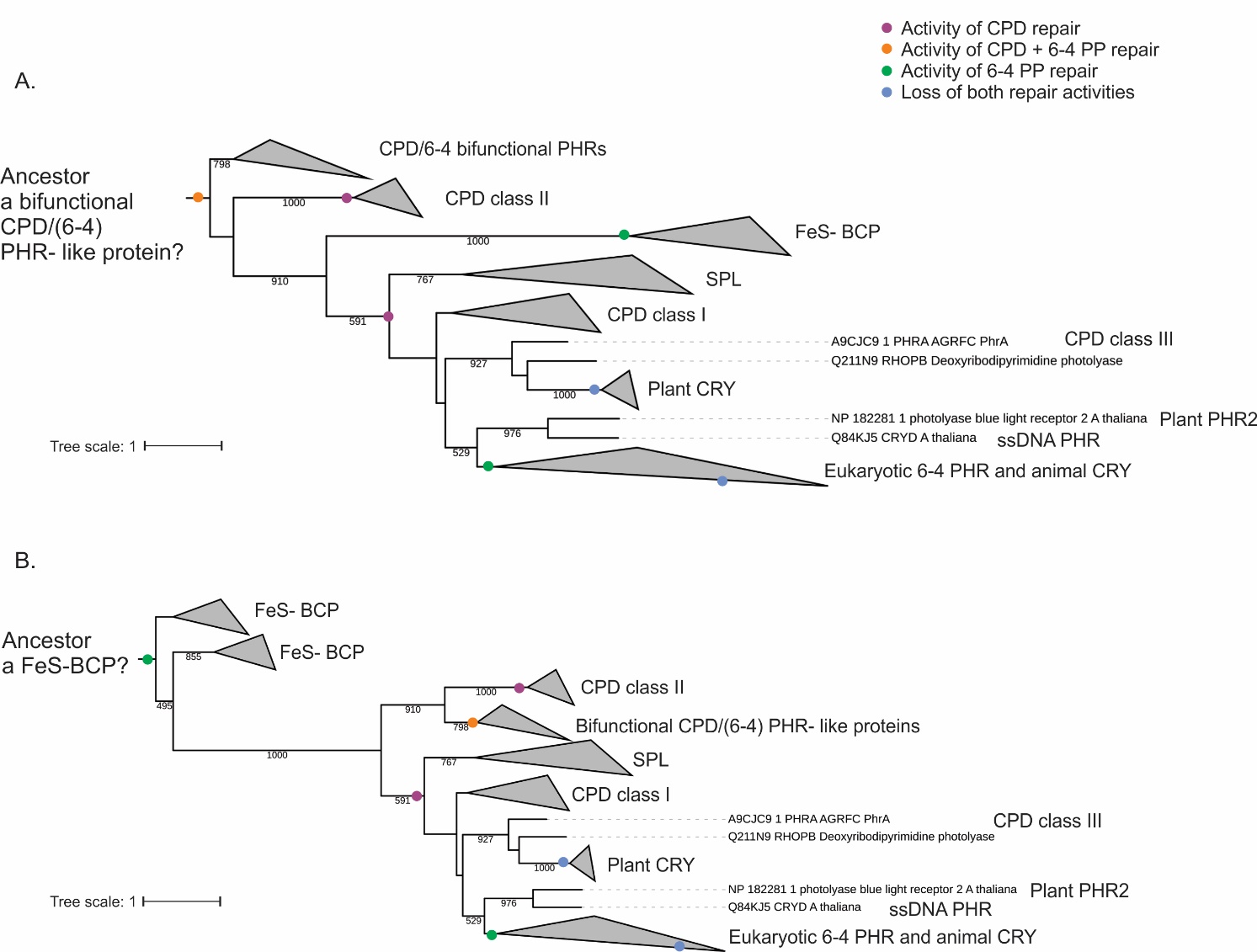
**

**Figure S3. Other possible evolutionary pathways of CRY/PHR family.** The earliest branching lineage of CRY/PHR family might be (*A*) bifunctional CPD/(6-4) PHR- like protein, (*B*) a 6-4 photolyase with an iron–sulfur cluster like a FeS-BCP. Proposed major evolutionary events regarding DNA damage repair activity are indicated by colored dots. Maximum-likelihood probabilities of 1000 replicates are adjacent to each internal node. Node support below 400 are not shown.

**
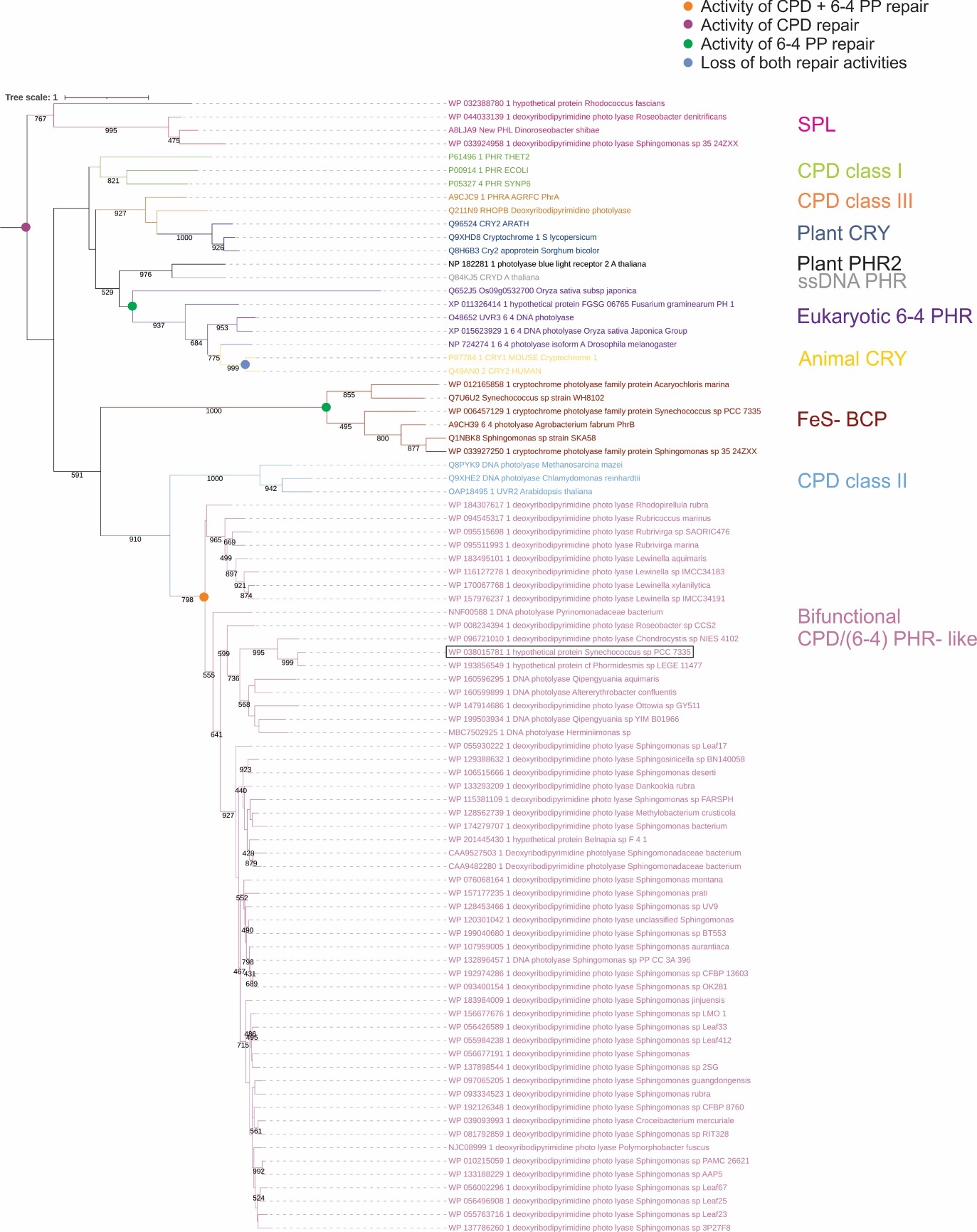
**

**Figure S4. Possible evolutionary pathways of CRY/PHR family.** Phylogenetic tree with a CPD photolyase with an iron–sulfur cluster (SPL) as the earliest branching lineage. Proposed major evolutionary events regarding DNA damage repair activity are indicated by colored dots. Maximum-likelihood probabilities of the node support below 400 are not shown. Bootstrap values of 1000 replicates are adjacent to each internal node. Position of SyCPD/(6-4) PHR- like is indicated with a rectangle.

**A.**

-10

-35

TTACCAGAACAACAAACACAATCACAATTGCCTTCTTGAAGCGTATGCATCCGCGCTTTG

CTATACAGGCGCTTACAGACAAAAGTAAAAAGTAAGTAGGAAGGATGATGCTCTGGGCAC

CCAGAATCTTCTAATGTTTGAATAAAATAGCGCTGTTTGATTCGAAGGACTTATTTCTTC

TTGATTGACGCTGCTGTTTTGATCAGACCCTTACCGTTAGCGGATAGCAATTGATGAGCG

CTGTACAAGTTGTGTGGTTCAAAAAAGACTTTCGACTCTTCGATCATCGCTGCTTGACGT

TCGCTGCGCAAAAGGGACCGGTTCTACCGCTTTATGTTGTCGAGCCAGACTACTGGGCAC

TAGAAGATACGAGCTATCGGCAGTATTTGTTCCTCAAGGGCTGTGTAGAAGAAACGATTG

AGGCATTAGAAGAGCTGGGTGGGTCGATGGCGATCGCCTCTGGTGATGTAGTAGAGGTGC

TCAGTCGACTCCAGAAAACATACGGCAATATCGAGCTTTGGGCACATCAGGAAACAGGCA

ACAACTGGACGTTTCAACGGGATAAAGCGGTTAGAAAGTGGTGCCAGCAAGCAGGGGTAG

CCTTCCATGAGCCGCTTCAGTTTGGCGTATGGCGAGGCTCCAAAATAAACCGCGATCACT

GGGCTAAGCAGTGGGATTCGCTGATGGCAGAACCCATCGCATCACTGCCAGAAAGCATCT

CTTTTGTAGCGCATTCTGAAGATAGAACGATGCCATCGCCCGAAGTACTAGGGCTGAAGC

ACGATGGCATCACCGCACTGCAGCCACCCGGTAGAAACGCAGCGCTTCAGGTTCTAAGCA

GTTTTTTGTACGAACGCGGCGAGAGATATCGTACAGATATGTCTTCGCCTGTGACTGGTG

AAAGTGGCTGCTCTAGGCTTTCTCCATACCTGGCCTACGGTGCGATCTCCATGCGCGAAA

CCTATCAAGCCACTCAAAAAAGAATTGCCGAAGTCGCTGCTATGCCCAAAGCAGAAAGGG

GGACCTGGGCCGGATCGCTCTCTTCTTTTGTGGGCCGTCTGCACTGGCACTGTCATTTCA

CTCAAAAGCTAGAGCTAGAGCCTGAACTTGAGTGGCTACCCATGGCAAGAGCCTATACAG

GCATCCGAGACGACGGCGACCATGCAATTAAGCTACGAGCGTTTGCAGAAGGTCAAACTG

GCTACCCCTTTGTTGATGCCTGTATGCGTTACCTGCGCGCGACCGGTTGGATTAACTTCC

GGATGCGAGCGATGCTGATGAGCTTTGCTAGCTACGACCTATGGCTGCCCTGGCAAAAGA

GTGGCGATGTTCTAGCCAGGCTTTTCACCGATTATGAGCCTGGCATCCACTGGCCTCAAT

CTCAGATGCAATCAGGCGTCACAGGTATCAACGCTATCCGGATCTATTCACCTATCAAAC

AAGGGCTAGACCAAGATATAGAAGGGACGTTTACCCGCCAGTGGGTTCCAGAACTCGCCG

CACTACCTGACGAAATACTACAGACACCTTGGCTCTTAGAAGGAGAACTCTCGTATCCGC

CGCCCATTGTGGAACATAAAGAGGCTGCAGCCTTAGCAAAGTCTAGGCTCTGGGCAATCA

AGAAAACACCTGAAGCAAAGAAAGAAGCTGAGCAAGTATACGAAAAGCACGGATCTAGAA

AGAAACCTCGTCGCAGAACTTCTTCAAGAAAGGCTTCGGCTACTTCCGCTCAGCGCACAA

AAGCTAAGCGTACTTCAAAAGGCAAGGCAAGCGCACAAAAAACGACTGAAAAACTCCCTA

CAAAAGCTGCTCAGGAGGCTGCTC**ATG**TCAGT**TGA**TATCGGGTTCGCTCAAAGCAGCGGC

GATCTGCCTGATAGAGTGACTGTTGCGAATGATCAACCTGTTTTGGGAGAGGGCAACTAT

GTTCTAGTGATCCTCAACCGAGCCTTACGAGGTGAAGACAACCCGGTCATAGACGCAGCA

CACCATCACGCGCGGGCGCTGGATTTACCTGTTGTTGTTTACAGCCAGTTAGACGAGCAG

ATCGATTGGGCGAATGACCGTCTGTTCTACTTTGCTTTGGGTGCGTTTAGAGAATGTGCG

ATCGCCCTTAGAAAGCGCAAAATCCAGTGCGTACAGACTCTAGAGCGAAAGGATGAGGAA

GGGACTATGCGATCGCTGCTCGCCGAAGCCGTCGCCATCTATACCGACGAAGATCCTACC

TTTCGAGATAGGGCTAGATTAGACAAAATTCTAGCAACTGCCCATCAAACCGTATTCTTT

GTAGACGCCGCTAGACTTGTTCCTGTCCGCAAGCTGCCAAAAGGGCTCAAGACAACCCCT

GCTTTTAGAAAAGCGCACGGAGAGTTGAGAGAGCGTTATGCTGAGCTGCGAGCAGCGACC

CAAACCAGTCAGATCAAGGCACAAACGCTATCGCTACCAAACGAACACAACTTCGCAGAC

TACAGCGACGCAGCTCTCAAAGATCTGGTTTCACAGTGCGATATCAATCACAGTATCAGA

ATCTCGAAAGAGCACCCACCGACTCAAGCAGTCATAGAAGCTCGAGTCAAAATCCTAAAA

GAGAAAATCCTTGCCAAATACAAATGGATTAGAAACAACGCAGCACTAGAACATTCCACC

TCACAGCTATCCCCCTATCTACACTTTGGCATGATTAGCCCGCACCAGCTCCTAGCTGAA

ATTGAATCTTCAGAGGTTCCCAAAACTTACACCTGGAAATTTCGAGACGAATTCCTAACC

TGGCGAGAATGGTCCCACTATCAGGCCTTCTACGTTCCTAATCTGCATGAATACAGGGCC

TTACCAGACAAAGCAAAAGAGACGCTAGAGGCTCATGCAGGCGATCCACGCCCCGAGCTG

CAATCAGATGAAGACATTTTGAACGCTCGCACGTCCGATAGTACCTGGAATGCTGCTCAA

AACGAGTGGTCGAATACGGGATGGCTACACAACAACCTGCGGATGTACTGGTCAAAACAA

ATCTTACGCTTTACCCGAACACCAGAAGCCGCCTGGGAAATGGCTTGCTACATCAATGAC

CATTTCTCTTTAGATGGCAGAGATCCTGCAACCTATGCCTCTATGCAGTGGGCCTTCGGT

AATGCGAAAAAAGGCTACAGCGAGAAGGAGATTTACGGATGGGTAGCACCGAAGTCTGAC

AGAGCTATCTTGAAAAGAGACGGCATGAAAGCATGGATTGAGCAAAGGCTATAGAGAGCC

AAATCCCTTCTTTTTTTTGTTCTTCTTTTTCTTCTTTCCACCGCCAGCGCCACGCCAACC

GGGCCTAGGCGCCCCACCGAAAGGATTTCTCCCCATCGGTCCGCCACCCATTGGACCGCC

GCCCATACCAGGAAAGCTACCGTCAAATCCTGGTAGCCCCATATTTCCTTGGCCCATTTG

CTGCATGAGTCCGCGCATCCGCTGAAAATCACTCACCAGCTTGCCGACATCCTTCTCGGC

ATAGCCAGCACCCTTTGCCACCCGCCGCCGCCGACTAGGCGAACCGGACAACACTTCTGG

GTTCTGCCGTTCTTCCATCGTCATCGAATTGATCATGGCCTCACAGCGCTTTAGCTGCTG


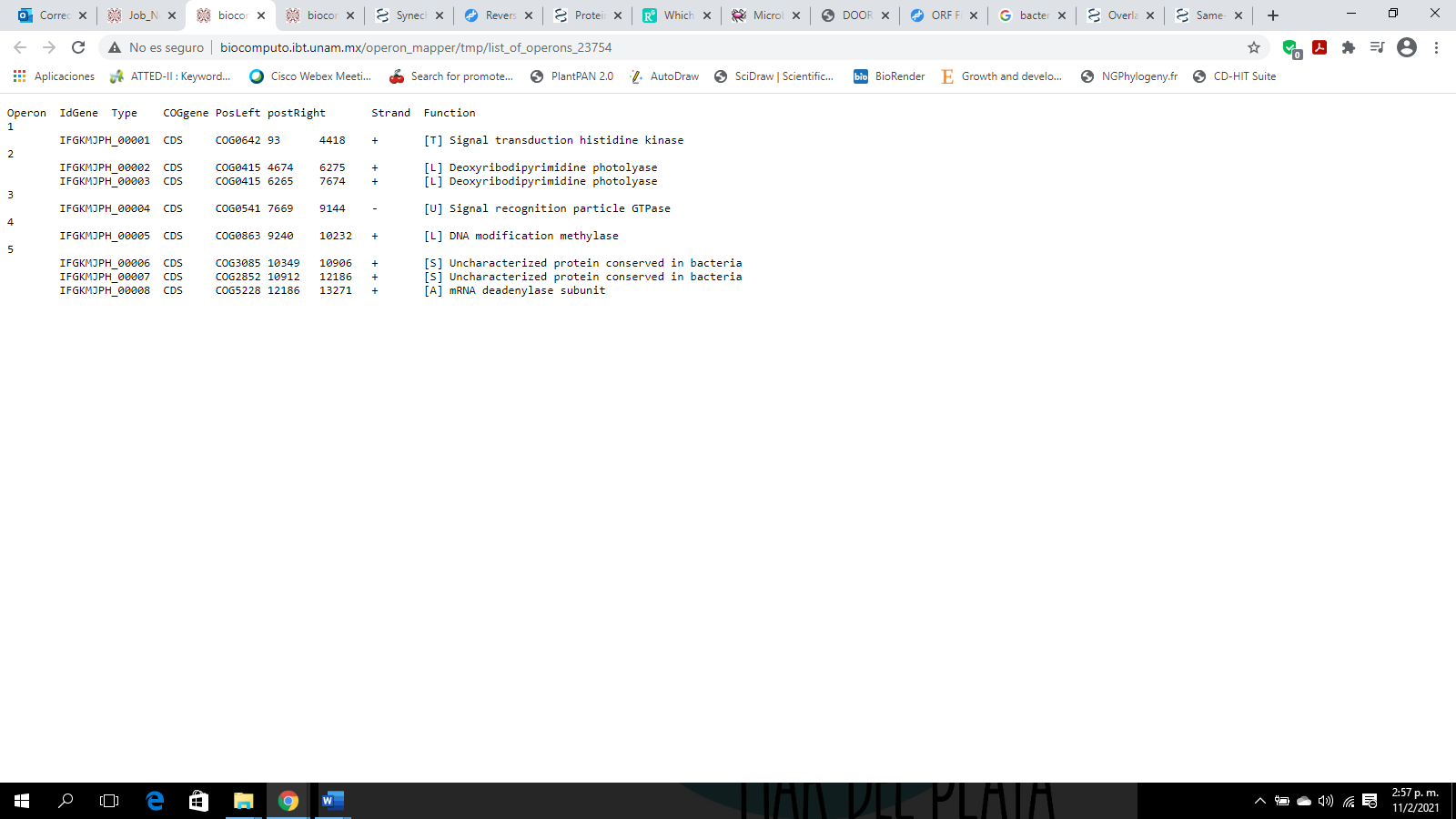
**B.**

**Figure S5. Genetic organization of the PHRs operon.** **A.** In blue, the PHR gene and in dark blue the bifunctional CPD/(6-4)- PHR. In pink, the overlapping region between both genes. In grey, the Shine Dalgarno sequence. The ATG from downstream gene and TGA from upstream gene are shown in bold. Underlined, primers used for RT- qPCR analysis. -35 and -10 boxes are indicated at the promoter region. In yellow and brown, the CRP and IHF transcription factors potential binding sites respectively. **B.** Operon mapper prediction of prokaryotic operons. IFGKMJPH_00002 corresponds to PHR and IFGKMJPH_00003 to SyCDP/(6-4) predicted bifunctional PHR.


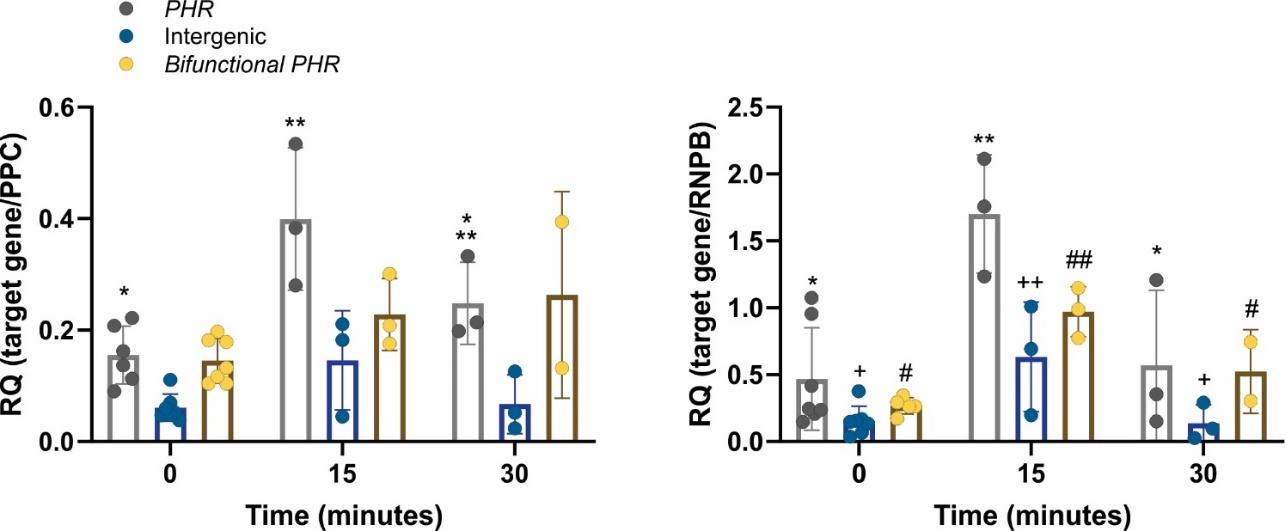


**Figure S6.** **Operon expression analysis under UV-B treatment by RT- qPCR.** *PHR* (grey), *SyCPD/6-4 PHR- like* (yellow) and the intergenic region (blue) transcript levels under control or UV-B treatment were evaluated by RT-qPCR. Results were expressed as 2^-(∆Ct)^ using as normalizer PPC (left panel) or RNPB (right panel). The One- way ANOVA test was used to determine differences for *PHR* (*), the intergenic region (+) and *bifunctional CPD/(6-4) PHR-like* (#) transcript levels at different time treatments (p <0.05). Equal symbols represent no statistical differences.
